# Real-world use of chemotherapy for Kaposi’s sarcoma in a large community-based HIV primary care system in Kenya

**DOI:** 10.1186/s12885-019-6506-3

**Published:** 2020-01-29

**Authors:** Esther E. Freeman, Naftali Busakhala, Susan Regan, Fredrick Chite Asirwa, Megan Wenger, Divya Seth, Khatiya Chelidze Moon, Aggrey Semeere, Toby Maurer, Kara Wools-Kaloustian, Ingrid Bassett, Jeffrey Martin

**Affiliations:** 1 Department of Dermatology, Massachusetts General Hospital, Harvard Medical School, Bartlett Hall 6R, 55 Fruit Street, Boston MA, MA 02114 USA; 2 AMPATH, Moi University, Eldoret, Kenya; 3 Indiana University, Indianapolis, Indiana, USA; 4 University of California, San Francisco, USA; 5 Infectious Diseases Institute, Makerere University, Kampala, Uganda

**Keywords:** Kaposi’s sarcoma, HIV-associated malignancy, Chemotherapy, Cancer care

## Abstract

**Background:**

Kaposi’s sarcoma (KS) is one of the most common HIV-associated malignancies in sub-Saharan Africa. Worldwide, the availability of antiretroviral therapy (ART) has improved KS survival. In resource-rich settings, survival has also benefited from chemotherapy, which is widely available. Little is known, however, about the epidemiology of chemotherapy use for HIV-associated KS in resource-limited regions such as sub-Saharan Africa.

**Methods:**

We identified all patients newly diagnosed with HIV-related KS from 2009 to 2012 in the 26-clinic AMPATH network, a large community-based care network in Kenya. We ascertained disease severity at diagnosis, frequency of initiation of chemotherapy, and distribution of chemotherapeutic regimens used. Indications for chemotherapy included AIDS Clinical Trial Group T1 stage and/or “severe” disease defined by WHO KS treatment guidelines.

**Results:**

Of 674 patients diagnosed with KS, charts were available for 588; 61% were men, median age was 35 years, and median CD4 at KS diagnosis was 185 cells/μl. At time of diagnosis, 58% had at least one chemotherapy indication, and 22% had more than one indication. For patients with a chemotherapy indication, cumulative incidence of chemotherapy initiation (with death as a competing event) was 37% by 1 month and 56% by 1 year. Median time from diagnosis to chemotherapy initiation was 25 days (IQR 1–50 days). In multivariable regression, patients with > 3 chemotherapy indications at time of diagnosis had a 2.30 (95% CI 1.46–3.60) increased risk of rapid chemotherapy initiation (within 30 days of diagnosis) compared to those with only one chemotherapy indication (*p* < 0.001). Initial regimens were bleomycin-vincristine (78%), adriamycin-bleomycin-vincristine (11%), etoposide (7%), and gemcitabine (4%).

**Conclusions:**

A substantial fraction of patients with KS in East Africa are diagnosed at advanced disease stage. For patients with chemotherapy indications, nearly half did not receive chemotherapy by one year. Liposomal anthracyclines, often used in resource-rich settings, were not first line. These findings emphasize challenges in East Africa cancer care, and highlight the need for further advocacy for improved access to higher quality chemotherapy in this setting.

## Introduction

In many areas of Africa affected by the HIV epidemic, HIV-associated malignancies are among the most common cancers in the overall population [1]. For example, despite growing availability of antiretroviral therapy (ART), Kaposi’s sarcoma (KS), as of 2018, remains the most incident cancer in many parts of eastern and southeastern Africa, including Malawi, Mozambique, Uganda, and Zambia [2]. Not only is KS common, but survival in these settings is also very poor. Our work across five African countries found that 45% of KS patients were lost to care by 2 years [3], and, once those patients were tracked, corrected mortality was 54% at 3 years [4]. One year survival after diagnosis of HIV-related KS in sub-Saharan Africa is estimated to be between 60 and 76%, [4–8] though slightly higher in a tightly monitored randomized controlled trial setting [9]. In contrast, in resource rich settings such as the U.S. and Europe, one year survival is 80–95% [10, 11].

Much of the explanation given for the high mortality of HIV-related KS in Africa has been attributed to advanced stage at presentation, but the role of therapy must be considered as well. There are two established therapies: ART and chemotherapy. ART is indicated for all HIV-infected patients with KS regardless of cancer severity [12], and can be effective alone as treatment in early stage disease. Indications for when to use chemotherapy are less clear. International guidelines vary slightly on when to begin chemotherapy [12, 13], but do agree that chemotherapy is indicated for all but early stage KS. In resource rich settings, liposomal anthracyclines (e.g. Doxil) or paclitaxel are recommended first line agents, based on efficacy [14]. Much less is known is about the use of chemotherapy in resource-limited settings such as sub-Saharan Africa, where cancer treatment facilities may have constrained resources and limited formularies. Most of the limited data on chemotherapy use for KS in sub-Saharan Africa either comes from clinical trials [15, 16] or from specialized, intensive treatment centers operated in collaboration with major donor agencies [17, 18]. Lacking comprehensive population-level data on real-world chemotherapy use in Africa, it is difficult to assess how much poor survival of KS is impacted by advanced stage presentation versus lack of access and effectiveness of available chemotherapy.

To address our limitations in knowledge about the epidemiology of chemotherapy use for HIV-related KS in Africa, we investigated all newly diagnosed cases of KS in a community-based HIV care program in East Africa. We determined the prevalence of indications for chemotherapy at the time of diagnosis, frequency of initiation of chemotherapy, drug regimens used, and factors associated with chemotherapy initiation.

## Methods

### Overall design

Via medical record review, we identified all patients newly diagnosed with HIV-related KS from 2009 to 2012 in the Academic Model Providing Access to Healthcare (AMPATH) consortium in Kenya. We determined indications for chemotherapy at the time of KS diagnosis (as detailed in *Measurements* below). In a cohort analysis, we then observed subsequent clinical course, including any therapy received, for 12 months following KS diagnosis.

### Study population

To identify all KS cases over the specified time period, we reviewed the clinic network’s electronic database as well as pathology records, and then followed up with detailed paper chart review. We included all patients living with HIV > 18 years old receiving their care at AMPATH if they received a clinical or biopsy-confirmed diagnosis of KS during this time. At the time of this study, AMPATH provided care to approximately 160,000 patients with HIV through 26 major clinical care sites. There is an established KS biopsy service, which is free of charge. However, biopsy diagnosis was not universally standard of care at the time of the study, and patients were allowed to be treated based on clinical grounds alone when a biopsy was not done. ART is also free of charge. For patients needing chemotherapy, this treatment is provided by the Oncology Department at a tertiary care hospital in Eldoret, Kenya, as well as smaller outreach clinics. At the time of the study, chemotherapy and associated fees were paid for by the patient. All patients provided written consent for data derived from their care at the participating clinic sites to be used for purposes of research through International Epidemiology Databases to Evaluate AIDS (IeDEA).

### Measurements

We ascertained KS disease severity at time of diagnosis and subsequent KS-specific treatment and survival through chart review. HIV/primary care charts, oncology charts, chemotherapy infusion records, and inpatient charts were retrieved for each participant. Paper charts were sought on three separate occasions before being deemed unobtainable. Record abstraction was performed by trained clinical officers using a structured chart review form. Double data entry was performed, and differences were reconciled by the principal investigator (PI).

KS severity at time of diagnosis was based on any symptom recorded within the first 30 days of KS diagnosis. Information on weight loss, diarrhea, night sweats, fever, Eastern Cooperative Oncology Group (ECOG) performance status, extent of cutaneous KS, lymphadenopathy, oral disease, GI KS, pulmonary KS, ulceration, and edema was extracted from the charts. We considered chemotherapy indications to be the presence of at least one AIDS Clinical Trials (ACTG) T1 qualifying criteria (tumor-associated edema or ulceration, extensive oral KS, or non-nodal visceral KS) [19] or the presence of at least one World Health Organization (WHO) KS Treatment Guidelines “severe disease” qualifying symptom (symptomatic visceral disease, extensive oral KS that interferes with swallowing, painful/disabling tumor-associated edema or ulcerated tumors, or life-threatening/functionally disabling disease) [12]. Demographic information, CD4 count, and use of antiretroviral therapy was extracted from AMPATH’s electronic database. KS-specific treatment with chemotherapy was ascertained through the chemotherapy infusion record, and charts were additionally reviewed for other forms of treatment such as injections, surgery, or radiation. Survival was initially determined through medical record review and, subsequently, for those who became lost to follow-up, updated through intensive community field tracking, which has been described elsewhere [3].

### Statistical analysis

Time zero was set as the earliest date the clinician had information about the KS diagnosis and therefore could potentially act on it. When a biopsy was performed, the date of the receipt of those results to the clinician (taken to be 10 days after the biopsy was performed accounting for local time needed to process and communicate results of the biopsy) was used for time zero. If a patient had both a clinical diagnosis date and a biopsy diagnosis date, the earlier of the two dates was used to establish time zero. Observation was continued from time zero to the initiation of chemotherapy or death for 12 months. Patients not known to be dead or to have started chemotherapy were administratively censored at last known date alive.

Cumulative incidence of chemotherapy initiation with death as a competing event was calculated using the Aalen-Johansen estimator [20]. Patients who were still alive at the end of observation time who had not received chemotherapy and had not died were administratively censored at last known date alive. We used binomial log-linear regression [21, 22] to evaluate the independent association between exposures at time of KS diagnosis (age, sex, KS disease severity, CD4 count, and socio-economic indicators) and the outcome of rapid chemotherapy initiation, defined as chemotherapy initiation within 30 days of KS diagnosis.

## Results

### Characteristics of the study population

Of 674 patients diagnosed with KS during the study period, 588 (87%) had charts available for extraction and were included in the study. The majority of diagnoses were made on clinical grounds alone, while 266 (45%) had biopsy-confirmed KS. Most (61%) were men and median age was 35 (Table 1). Almost all patients (90%) had completed at least primary education level, with few (4%) reaching tertiary education. Patients lived in both rural and urban settings, with travel times to clinic ranging from a few minutes to over 2 h. As a measure of socioeconomic status, the majority of patients (87%) did not have running water piped into their homes.

**Table 1:** Characteristics of HIV-infected patients newly diagnosed with Kaposi’s sarcoma between 2009 and 2012 in a large community-based primary care network in Kenya

Characteristic	Median (Interquartile range) or Percentage (*N* = 588)
Age, years	35 (30–42)
Male gender	357 (61%)
Documented Symptoms at KS diagnosis	
Weight loss (self-reported)	139 (24%)
Diarrhea (self-reported)	47 (8%)
Night sweats	56 (10%)
Fever (self-reported or measured)	107 (18%)
ECOG performance status *Mean (SD)* ^a^	1.34 (0.80)
ECOG > 1	72 (12%)
Extent of KS on skin	
Localized to one anatomic area	115 (20%)
More than localized	257 (44%)
Lymphadenopathy	72 (12%)
KS Lesions in oral cavity	176 (30%)
Interfering with swallowing	30 (5.1%)
GI KS suspected/confirmed	30 (5.1%)
Pulmonary KS suspected/confirmed	51 (8.7%)
> 1 Ulcerated KS lesion	69 (12%)
Edema *N*	247 (42%)
Interfering with function	26 (4.4%)
CD4+ T cells, count/μl ^b^	184 (68–350)
< 50	71 (12%)
51–100	48 (8.2%)
101–200	79 (13%)
201–350	80 (14%)
> 350	92 (16%)
Antiretroviral therapy (ART)	
On ART at time of diagnosis	177 (30%)
Days on ART among patients on ART at time of KS diagnosis	119 (42–539)
On ART > 60 days prior to KS diagnosis	112 (19%)
Chemotherapy indication	
ACTG T1	333 (57%)
WHO “Severe KS”	92 (16%)
Education	
None	56 (10%)
Primary	346 (62%)
Secondary	135 (24%)
Tertiary	25 (4.4%)
Travel time to clinic	
< 30 min	132 (23%)
30–60 min	193 (34%)
1–2 h	135 (23%)
> 2 h	115 (20%)
Water piped into home	74 (13%)

^a^ECOG = Eastern Cooperative Oncology Group performance status

^b^CD4 count proximal to diagnosis was defined as the closest CD4 count to the date of KS diagnosis within a window from 30 days prior to diagnosis or up to 60 days after the diagnosis date. There was no proximal CD4 count available on 218 of the participants

Median proximal CD4 count was 184 cells/mm^3^. Plasma HIV RNA levels were not available because viral load was not routinely collected as part of clinical care in Kenya between 2009 and 2012 due to cost in this setting. A minority of patients, 177 (30%) were on ART at time of KS diagnosis, with 112 (19%) on ART for > 60 days at time of diagnosis. The ART regimen administered prior to 2011 in Kenya included stavudine (d4T) or zidovudine (AZT), plus lamivudine (3TC) and efavirenz (EFV) or nevirapine (NVP) [23]. In 2011, new national guidelines were published recommending tenofovir (TDF) or zidovudine (AZT) in combination with lamivudine (3TC) and efavirenz (EFV) or nevirapine (NVP) as the preferred first line treatment [24].

### Severity of KS at time of Cancer diagnosis

The most common symptoms at time of KS diagnosis were non-localized skin disease (e.g. skin disease that involved more than one anatomic location), edema, and oral lesions. Relatively few had suspected or confirmed gastrointestinal or pulmonary KS (5 and 9%, respectively, Table 1). Collectively evaluating ACTG tumor status (tumor associated edema/ulceration, oral KS, and visceral KS), the majority (57%) of patients had ACTG T1 disease. The most common T1 qualifying criteria was edema (42%). Few patients were noted to have WHO KS Treatment Staging “severe” disease, since functional impact of symptoms (such as whether oral disease was interfering with swallowing) was not routinely noted in charts. At least one chemotherapy indication (taken to be either ACTG T1 or WHO severe disease) was present in 58% of patients at time of KS diagnosis; 22% had more than one chemotherapy qualifying criteria. In univariate analysis, being on ART at time of diagnosis was associated with diagnosis at a less advanced KS disease stage (*p* = 0.004).

### Chemotherapy initiation

**Fig. 1:**
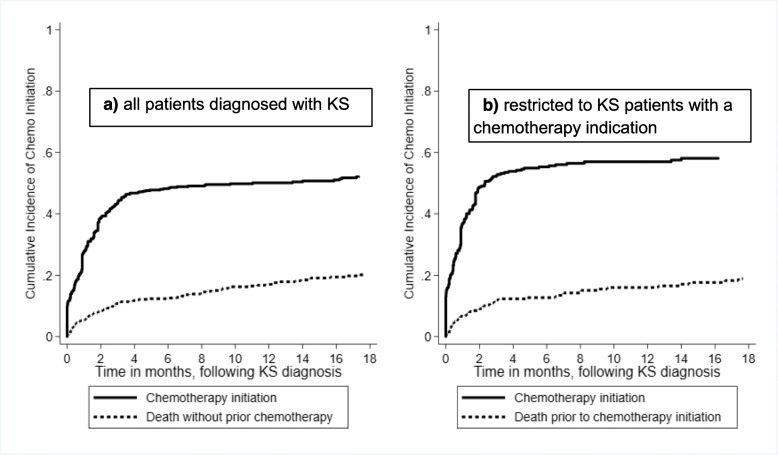
Cumulative incidence of chemotherapy initiation after KS diagnosis among HIV-infected patients newly diagnosed with Kaposi’s sarcoma between 2009 and 2012 in a large community-based primary care network in Kenya. Death prior to chemotherapy is accommodated as a competing event. **a** all patients diagnosed with KS **b** restricted to KS patients with a chemotherapy indication

For the entire cohort, the cumulative incidence of chemotherapy initiation (with death as a competing event) was 27% by 1 month, 43% by 3 months, 48% by 6 months, and 50% by 1 year (Fig. 1a). Death prior to chemotherapy initiation was 0.5% by 1 month, 11% by 3 months, 12% by 6 months, and 17% by 1 year. Restricted to patients with a chemotherapy indication at time of diagnosis, the cumulative incidence of chemotherapy initiation was 37% by 1 month, 52% by 3 months, 55% by 6 months, and 56% by 1 year (Fig. 1b). Median time to chemotherapy initiation was 25 days (IQR 1–50 days) in those with a chemotherapy indication. For this same group of patients (those with a chemotherapy indication at KS diagnosis), death prior to chemotherapy initiation was 6% by 1 month, 11% by 3 months, 12% by 6 months, and 16% by 1 year. The percentage lost to follow up without known outcome was extremely low (2.6%), due to intensive field tracking of lost participants in a prior study, which obtained updated vital status on lost individuals [3, 4].

The most common first-line chemotherapy regimen in this Kenyan cohort was bleomycin-vincristine (BV), at 78% (*n* = 228). Of the remaining patients, 11% received doxorubicin in addition to BV, while 7% received etoposide and 4% received gemcitabine. No patients received paclitaxel or liposomal anthracyclines as first line chemotherapy. The most common adverse effects included peripheral neuropathy (43.8%), neutropenia (25%), and gastrointestinal (GI) upset (25%).

### Determinants of chemotherapy initiation

**Fig. 2:**
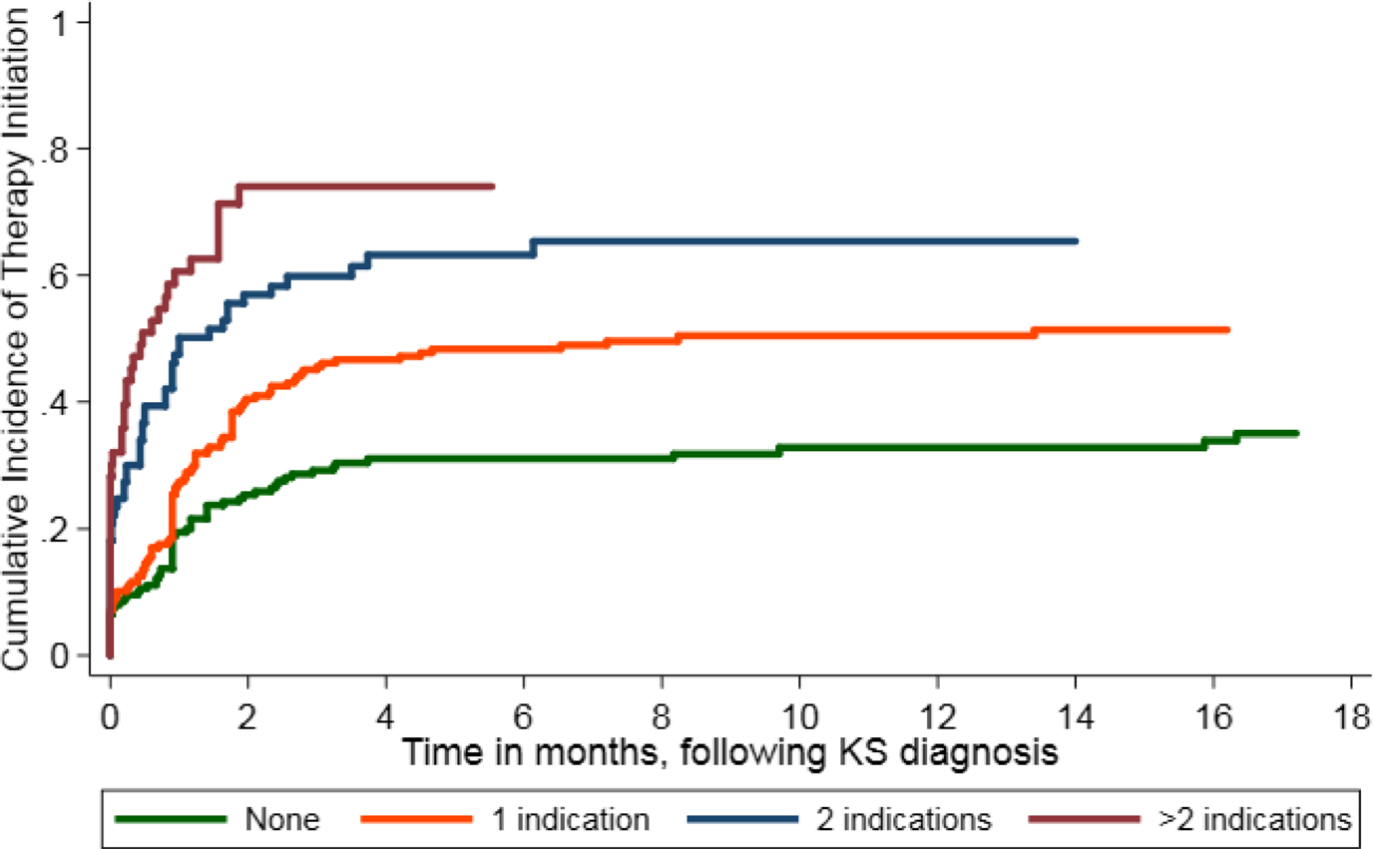
Cumulative incidence of chemotherapy initiation, according to number of indications, among HIV-infected patients newly diagnosed with KS between 2009 and 2012 in a large community-based primary care network in Kenya. Death prior to chemotherapy is accommodated as a competing event. An indication for chemotherapy includes either ACTG T1 disease or WHO Treatment Guidelines severe disease, recorded within the first 30 days of KS diagnosis. (Curves end when there is no more observation time of patients in that sub-group (e.g. due to death))

Cumulative incidence of chemotherapy initiation stratified by severity of disease, demonstrated that at 1 month 59% of the sickest patients who had > 2 chemotherapy indications had started chemotherapy, compared to 27% in the same time period in those with only 1 indication (Fig. 2). Similarly, at 6 months, cumulative incidence of chemotherapy initiation was 74% in those with > 2 chemotherapy indications, and 48% in those with only 1 chemotherapy indication. There were some patients that did not have a chemotherapy initiation at time of diagnosis who did ultimately receive chemotherapy (30% by 6 months). Some of these patients may have subsequently developed a chemotherapy indication after the initial 30 days resulting in eventual chemotherapy initiation, however we were not able to assess KS severity as a time-varying covariate.

We present two adjusted models for determinants of chemotherapy initiation within 30 days of KS diagnosis (Table 2). Model 1 includes age, gender, chemotherapy indications, and CD4 T+ cell count. Model 2 additionally includes socio-economic indicators of education, travel time to clinic, and the presence or absence of water piped into the home. Both models yielded similar results. The presence of chemotherapy indications (e.g. the stage of disease severity at time of diagnosis) was strongly associated with chemotherapy initiation within 30 days of KS diagnosis. The sickest patients, with three or more chemotherapy indications at time of diagnosis, had a 2.30 (95% CI 1.46–3.60, Model 1) times increased risk of rapid chemotherapy initiation compared to those with only one chemotherapy indication (*p* < 0.001). Similarly, in a sensitivity analysis restricted to patients with biopsy proven KS only, patients with three or more chemotherapy indications had a 2.12 (95% CI 0.93–4.86, Model 1) times risk of rapid chemotherapy initiation, though this did not quite reach statistical significance due to the smaller sample size of patients that had biopsy-proven disease (*p* = 0.07).

**Table 2:** Unadjusted and adjusted determinants of chemotherapy initiation within 30 days of Kaposi’s sarcoma (KS) diagnosis among HIV-infected patients newly diagnosed with KS between 2009 and 2012 in a large community-based primary care network in Kenya

	Unadjusted		Adjusted Model 1^a^		Adjusted Model 2^a^	
Characteristic	Risk Ratio (95% CI)	*P* value	Risk Ratio (95% CI)	*P* value	Risk Ratio (95% CI)	*P* value
Age, years						
≤ 30	Ref.		Ref.		Ref.	
30–34	1.02 (0.72–1.45)	0.908	1.04 (0.64–1.68)	0.872	1.00 (0.60–1.66)	0.997
35–39	0.66 (0.42–1.03)	0.068	0.72 (0.41–1.25)	0.242	0.62 (0.34–1.13)	0.119
≥ 40	0.81 (0.55–1.19)	0.285	0.85 (0.51–1.39)	0.511	0.83 (0.49–1.41)	0.489
Gender						
Women	Ref.		Ref.		Ref.	
Men	1.10 (0.82–1.49)	0.520	1.10 (0.75–1.62)	0.623	1.04 (0.69–1.57)	0.838
Chemotherapy indications						
1 chemotherapy indication	Ref.		Ref.		Ref.	
2 chemotherapy indications	1.75 (1.26–2.44)	0.001	1.82 (1.19–2.79)	0.006	1.86 (1.20–2.87)	0.005
≥ 3 chemotherapy indications	2.18 (1.58–3.01)	< 0.001	2.30 (1.46–3.60)	< 0.001	2.43 (1.51–3.91)	< 0.001
CD4+ T cells, count/μl						
> 350	Ref.		Ref.		Ref.	
201–350	1.55 (0.93–2.59)	0.091	1.61 (0.85–3.06)	0.143	1.54 (0.80–2.98)	0.200
51–200	1.65 (1.03–2.63)	0.036	1.74 (0.97–3.11)	0.063	1.86 (1.03–3.37)	0.040
< 50	1.11 (0.60–2.07)	0.731	1.04 (0.49–2.20)	0.913	1.04 (0.49–2.23)	0.909
CD4 unknown	1.06 (0.65–1.74)	0.720	1.07 (0.60–1.92)	0.820	1.03 (0.56–1.89)	0.921
Education						
None	Ref.				Ref.	
Primary	1.00 (0.55–1.80)	0.990			0.85 (0.39–1.85)	0.689
Secondary	1.18 (0.64–2.19)	0.590			1.09 (0.48–2.44)	0.842
Tertiary	1.31 (0.56–3.07)	0.540			1.20 (0.38–3.82)	0.760
Travel time to clinic						
< 30 min	Ref.				Ref.	
30–59 min	1.05 (0.67–1.65)	0.837			1.06 (0.60–1.87)	0.835
1–2 h	1.22 (0.78–1.91)	0.387			1.18 0.67–2.07)	0.567
> 2 h	1.42 (0.91–2.20)	0.119			1.37 (0.76–2.45)	0.295
Water piped into home						
No	Ref.				Ref.	
Yes	1.11 (0.74–1.68)	0.604			1.14 (0.65–1.97)	0.652

^a^all variables adjusted for all other variables in column

In the multivariate models, neither age nor gender were significant predictors of chemotherapy initiation. Restricting to biopsy proven KS cases did not change this result. None of the evaluated socioeconomic indicators (Model 2) were associated with chemotherapy initiation. Notably, there was limited variability in the socioeconomic status of patients using these Ministry of Health supported clinics; 72% either received no education or primary school only, and 87% did not have running water in their homes. Having a very low CD4+ T cell count < 50 was not associated with rapid chemotherapy initiation compared to those with the highest CD4 > 350. However, moderately low CD4+ T cell count of 51–200 was associated with a modest increase in chemotherapy initiation (relative risk 1.74 95% CI 0.97–3.11 in Model 1, 1.86 95% CI 1.03–3.37 in Model 2). Being on ART at time of diagnosis was not associated with chemotherapy initiation within 30 days of KS diagnosis.

### Joint outcomes of chemotherapy and survival over time

**Fig. 3:**
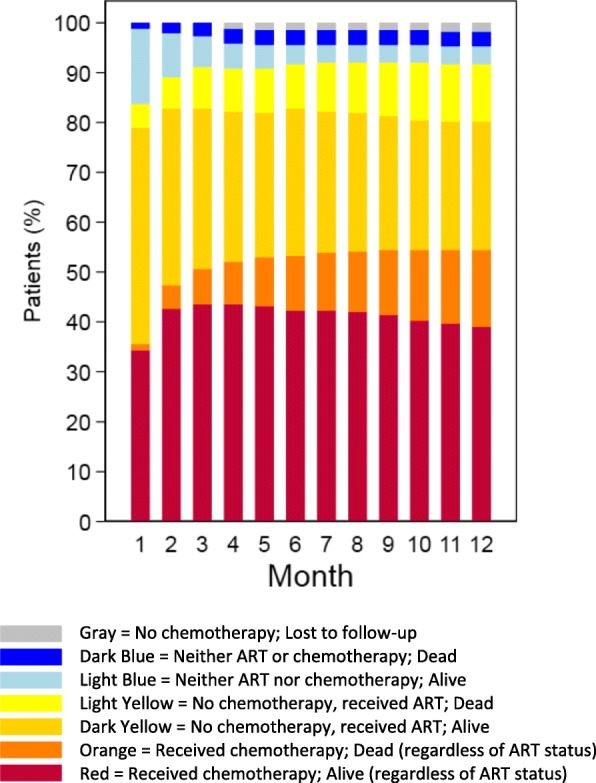
Status of chemotherapy and vital status by month since diagnosis of Kaposi’s sarcoma (KS) among HIV-infected patients newly diagnosed with KS between 2009 and 2012 in a large community-based primary care network in Kenya. The analysis is restricted to patients with a chemotherapy indication at time of KS diagnosis

Among patients with an indication for chemotherapy at time of KS diagnosis, we evaluated the joint occurrence of chemotherapy and death over time (Fig. 3). By 12 months, 47% had initiated chemotherapy and 32% had not initiated chemotherapy but had received ART alone. Of those who received chemotherapy, 72% were alive at 12 months and 28% had died; of those receiving ART only, 69% were alive and 31% had died. Overall, of all patients with a chemotherapy indication, 15% of patients died without receiving chemotherapy, either while on no treatment or while on ART alone.

## Discussion

KS mortality in sub-Saharan Africa is high, which led us to investigate the use of chemotherapy for treatment of KS in this setting. Among patients in a primary care HIV cohort in Kenya, just over half of patients with KS who had an indication for chemotherapy received it by the end of 1 year. Patients with the most severe disease were the most likely to receive prompt treatment within 30 days of KS diagnosis (relative risk 2.30; 95% CI 1.46–3.60). However, a substantial group of patients (44%), who seemingly had an indication for chemotherapy based on the severity of their KS disease, did not receive it within 1 year. Given the high mortality of KS in sub-Saharan Africa [2, 3], this considerable gap in treatment highlights a potential target for future interventions to improve survival.

Prior studies on KS treatment with chemotherapy in sub-Saharan Africa have focused on outcomes of particular chemotherapy regimens, [17, 18, 25–27] but have neither evaluated real-world delays in chemotherapy initiation, nor, perhaps most importantly, chemotherapy non-initiation in patients with chemotherapy indications. We know that there is often a significant delay between symptom recognition and successful initiation of cancer care in Africa [28]. Our results for KS echo data from other cancers in Africa, such as lymphoma and breast cancer, with similar delays [29] and non-initiation of chemotherapy [30]. A particular strength of our study comes from linking severity of disease at time of diagnosis with subsequent treatment course in a real-world setting.

Similar to other resource poor settings, most patients in this Kenyan setting were diagnosed with KS when their disease was already at advanced stage, and not all patients had histologically confirmed disease [8, 25, 31, 32]. We found that patients who were on ART for > 60 days prior to KS diagnosis were diagnosed at less advanced KS disease stage, which could either be due to the effects of ART itself (though we do not know who was virally suppressed), or due to the more frequent contact these patients had with the health system. This finding is in line with what has been reported for other co-morbid cancers and HIV [33, 34], although not universally [35]. This finding also reinforces that being on ART does not completely eliminate the risk of developing KS [36, 37].

The most common first line chemotherapy regimen in this setting was bleomycin plus vincristine. Liposomal anthracyclines (e.g., liposomal doxorubicin) and paclitaxel, which are first-line medications in resource-rich settings, were not used as initial therapy in any patient. Liposomal anthracyclines are recommended over BV when available in both the WHO and the National Comprehensive Cancer Network (NCCN) guidelines [12, 13], although these international guidelines were not yet in place during the time period of the study. Additionally, in sub-Saharan Africa, BV has recently been shown to be less efficacious than paclitaxel (in trial ACTG 5263, which was stopped early) for treating AIDS-related KS [38]. There has been increasing recognition for the need for highly efficacious chemotherapy in the African setting [39, 40], although more advocacy is desperately needed to decrease drug costs and improve accessibility. Improvement in the effectiveness of chemotherapy available to patients in low resource settings could improve survival beyond the direct effects of the drug. Specifically, peer to peer evidence of good outcomes may reduce fatalism and encourage patients to start and adhere to therapy, as occurred with ART when it became more widespread [41].

Patients with more chemotherapy indications (e.g. more advanced disease stage at time of diagnosis) are more likely to achieve rapid chemotherapy initiation within 30 days of diagnosis. That the most ill patients are being prioritized for chemotherapy is reassuring. The lack of impact of socioeconomic factors on chemotherapy initiation was initially surprising, since we hypothesized that patients who reside farther from clinic with less education and fewer resources would have a harder time accessing care. There are several possible reasons for this observation. This lack of effect may be due to the relative homogeneity of this population of care seekers, who are overwhelmingly economically disadvantaged and have at most a primary school education, where small variations in socioeconomic status may not impact access to chemotherapy. It is also possible that we need more sensitive socio-economic measures to delineate this effect. Alternatively, the community outreach programs of AMPATH, where chemotherapy is supplied to two other community clinics outside of the main hospital, may have helped to mitigate the effect of socioeconomic status on access to chemotherapy.

This study had several limitations. First, we identified people only after they had been diagnosed with KS. Therefore, we did not account for patients in the community who either did not seek care, or sought care but did not receive a formal KS diagnosis. Therefore, it may overestimate the true proportion of KS patients that received chemotherapy. Second, we were unable to locate paper charts for 13% of patients over the study period who were diagnosed with KS. These patients were excluded from the study since we were unable to ascertain whether or not they had received chemotherapy. Thirdly, severity of KS at time of diagnosis was limited to what clinicians recorded in the patients’ charts during routine clinical care. Record keeping was often incomplete. Absence of a symptom (e.g. a pertinent negative) was rarely recorded. Therefore, when a particular symptom was not documented, we made the assumption that the patient did not have that symptom. It is possible, therefore, that we underestimated the severity of disease at diagnosis. In particular, WHO severe disease was likely to be underestimated, since it was not common practice to record whether the disease was affecting the patient’s functional status.

Due to the retrospective nature of this chart review and incomplete paper records, we were not able to ascertain reasons that eligible patients did not start on chemotherapy. Literature suggests that for other cancers in sub-Saharan Africa affordability [42, 43], transportation, and fear, all may contribute to non-initiation [44], but prospective evaluation is needed to understand the differential contributions of these patient-level factors. Similarly, patients’ adherence to ART may influence the decision to start chemotherapy therefore should be investigated in future work as well. In addition, factors related to healthcare providers, rather than the patients themselves, such as provider non-recognition of disease severity, limited training, difficulty in obtaining pre-treatment tests, conflicting guidelines, or decisions made to prioritize end of life quality over treatment initiation [45–47], could all factor in to provider’s decisions not to start a patient on chemotherapy. In order to develop interventions to improve chemotherapy initiation in this setting, additional prospective evaluation of both these patient and provider level factors is needed.

Regarding generalizability, this Kenyan cohort consists of the general population receiving HIV primary care in a resource-poor sub-Saharan Africa setting. However, the AMPATH program does have access to enhanced oncology services, including a public cancer center with routine outreach clinics and an IeDEA-sponsored KS biopsy service. Therefore, both diagnosis and treatment in the setting may be enhanced compared to other public sector settings elsewhere in sub-Saharan Africa [17].

## Conclusion

In conclusion, we found that 44% of patients with KS in this Kenyan setting who may have needed chemotherapy at the time of diagnosis did not initiate this treatment. The subset of patients who successfully initiated chemotherapy did, for the most part, receive it in a timely manner. Due to the retrospective nature of this study, we could not further elucidate the reasons for treatment non-initiation, or distinguish between patient and provider factors. Prospective evaluation is needed to assess barriers to chemotherapy from both patient and provider perspectives, so that appropriate interventions can target treatment initiation. The chemotherapy regimens used at the time of the study, and what is still routinely available in Kenya, are not equivalent to liposomal anthracyclines, which are first line in resource-rich settings. International advocacy for more efficacious, affordable, and accessible chemotherapy in sub-Saharan Africa is critical, and improved outcomes could encourage other patients to seek out and initiate chemotherapy.

## Data Availability

The datasets used and analyzed during the current study are available from the corresponding author on reasonable request.

## Abbreviations

3TC: Lamivudine
ACTG: AIDS Clinical Trials
AMPATH: The Academic Model Providing Access to Healthcare
ART: Antiretroviral therapy
AZT: Zidovudine
BV: Bleomycin-vincristine
d4T: Stavudine
ECOG: Eastern Cooperative Oncology Group
EFV: Efavirenz
GI: Gastrointestinal
HIV: Human Immunodeficiency Virus
IeDEA: International Epidemiology Databases to Evaluate AIDS
KS: Kaposi’s sarcoma
NCCN: The National Comprehensive Cancer Network
NVP: Nevirapine
PI: Principal Investigator
TDF: Tenofovir
WHO: World Health Organization
